# Supplementary material for: Flame-Retarded and Heat-Resistant PP Compounds for Halogen-Free Low-Smoke Cable Protection Pipes (HFLS Conduits)
Source: Polymers (Basel). 2024 May 6;16(9):1298. doi: 10.3390/polym16091298 (PMC11085554; doi:10.3390/polym16091298)
Supplement: Supplementary file 1 [file polymers-16-01298-s001.zip › polymers-2958978-supplementary.pdf]

SUPPLEMENTARY INFORMATION

Table S1: Thermal properties of developed FR compounds and Industrial scale MBs. Comparison to reference PP and PP carrier.

Samples	TGA				DSC			
	$T_{5\%}$ (°C)	T_{d1} (°C)	T_{d2} (°C)	R (%)	OOT (°C)	T_c (°C)	X_c (%)	T_m^2 (°C)
**Lab-Scale Compounds**								
**PP**	391.7±0.0	433.5±1.2	*n.d.*	1.1±0.1	245.7±0.4	115.7±0.9	32.2±2.9	167.0±0.2
**FR1**	317.4±3.0	447.8±3.1	*n.d.*	2.5±0.3	255.0±3.4	108.8±3.1	36.8±0.5	170.6±2.0
**FR2**	351.7±6.7	431.3±7.7	*n.d.*	1.6±0.4	248.4±0.7	115.3±1.6	32.2±1.7	167.5±0.5
**Industrial Scale Masterbatches (MBs)**								
**PP carrier**	410.1±2.3	456.3±1.9	*n.d.*	0.0±0.0	*n.d.*	106.5±0.2	39.6±2.8	168.0±0.0
**MB1**	307.1±1.5	378.4±8.4	457.6±5.1	10.3±0.4	*n.d.*	104.7±1.9	39.3±5.9	160.5±2.1
**MB2**	314.6±0.1	328.0±0.2	475.1±2.9	29.5±0.6	*n.d.*	118.0±3.8	42.8±12.3	163.6±0.1

Table S2: Determined mechanical properties of the FR compounds prior to ageing.

FR Compounds	Tensile Tests			Impact Tests	
	σ_{max} (MPa)	ε_{max} (%)	E (GPa)	a_{iu} (kJ/m^2)	RSD (%)
**PP**	25.1±0.6	75.9±11.6	1.10±0.01	59.3±3.3	5.6
**FR1**	24.4±0.4	47.6±4.3	1.30±0.01	66.2±14.1	21.3
**FR2**	28.2±1.0	42.9±8.4	1.20±0.01	57.9±8.1	14.0

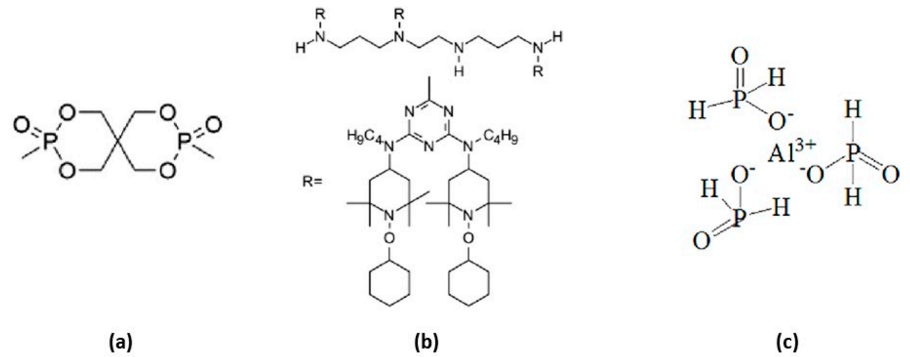

Figure S1: Chemical structure of the used additives. (a) ADD1: Cyclic phosphonate ester (Aflammit PCO 900), (b) ADD2: monomeric N-alkoxy hindered mine (Flamestab NOR116), (c) aluminum hypophosphite major compound of ADD3 and ADD4.

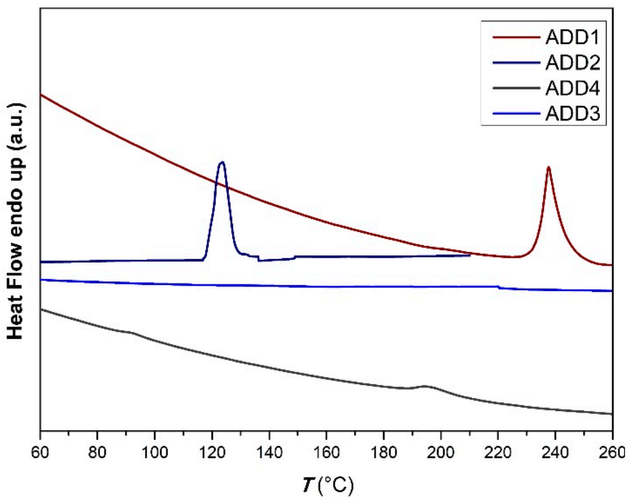

Figure S2: 1st heating DSC curves of the used additives.

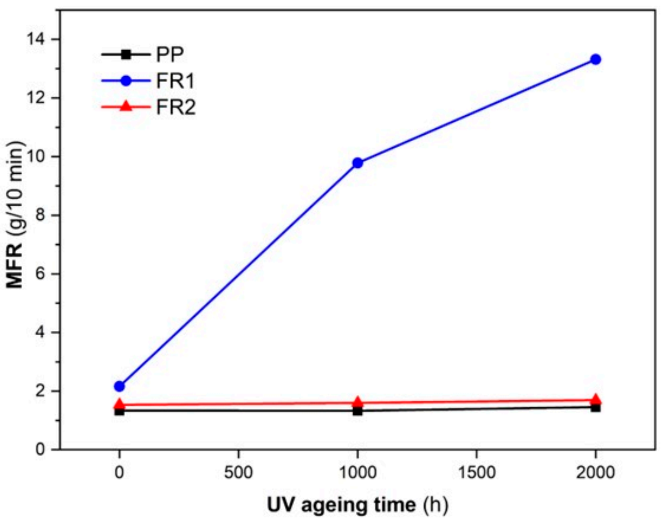

Figure S3: MFR of PP reference FR1 and FR2 during UV ageing tests.

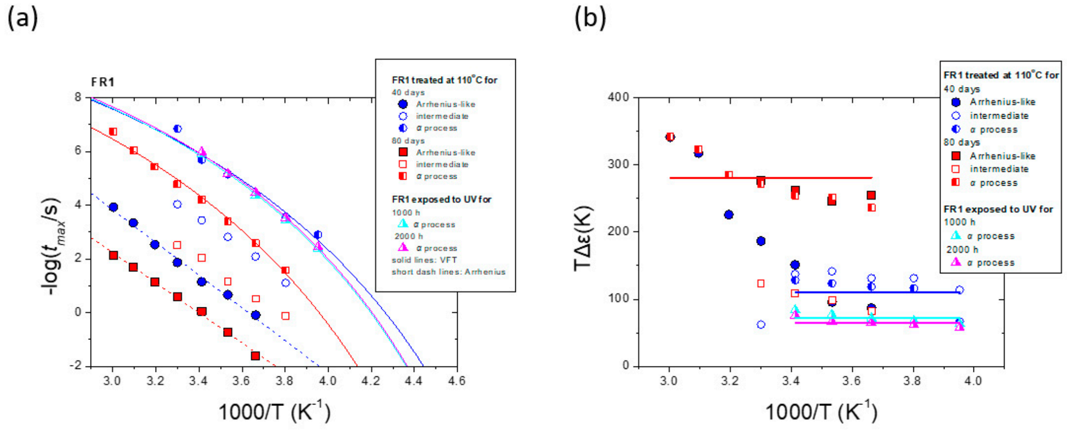

Figure S4: (a) Relaxation map for LA (red) and LA in P_2O_5 (blue). Filled circles correspond to α process, crossed-circles to dc-conductivity and yellow filled squares indicate the T_g at τ=100 s. Lines correspond to VFT fits of the experimental data. (b) Normalized Dielectric Strength ($T\Delta\varepsilon$) (top) and Parameters m, mn (bottom).

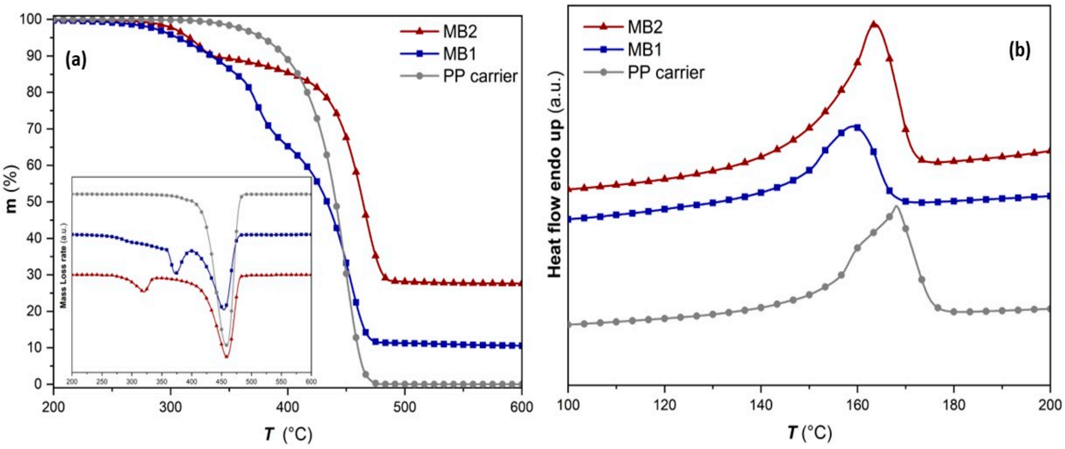

Figure S5: (a)TGA and (b) DSC curves of the industrial scale masterbatches MB1, MB2 and the PP carrier.
